# *“Some distance between us”*: a UK mixed methods study exploring experiences of remote care for eating disorders during COVID-19

**DOI:** 10.3389/fpsyt.2024.1383080

**Published:** 2024-06-06

**Authors:** Claire Murphy-Morgan, Richard Brown, Charlotte Love, Dawn Branley-Bell

**Affiliations:** ^1^ Department of Psychology, Northumbria University, Newcastle uponTyne, United Kingdom; ^2^ School of Psychology, Newcastle University, Newcastle uponTyne, United Kingdom

**Keywords:** eating disorders, eating distress, COVID-19, telehealth, eHealth

## Abstract

**Introduction:**

COVID-19 necessitated a rapid move from face-to-face services to remote care for eating disorders/eating distress (EDs). This study explores the advantages and challenges of remote care, identifying future implications for service provision. Remote care has been considered in the broadest of terms, including therapeutic care (e.g., Cognitive Behavioural Therapy, peer support, forums, one-to-one and group care options).

**Methods:**

Using a mixed methods approach, data were collected from 211 people with lived experience of EDs (PWLE), with and without formal diagnosis. 27 participants took part in semi-structured interviews/workshops and a further 184 participants took part via an online survey. Participants reported on their ED status, the impact of the pandemic on symptoms, the benefits, and challenges of remote care (and type of support accessed), and any reasons for not accessing support. Participants were invited to make future care recommendations.

**Results:**

ED symptoms were reported as worsening during the pandemic with contributing factors including isolation, lack of routine, negative emotions, and feeling like the external situation was outside of one’s control. Remote care was positively attributed to increased flexibility and facilitation of social connection. Identified barriers to access included lack of awareness about support availability, digital access/literacy, and competing commitments. Further challenges included approaches being perceived as too clinical (e.g., ED information and support presented using clinical language and/or limited to support within medical care settings, without acknowledging the broader context of disordered eating), uncertainty around remote care quality, and concerns that remote platforms may facilitate masking of symptoms. Participants reported distress caused by online platforms where self-view is the default during video calls. They expressed a need for more holistic approaches to remote care, including: “real stories” of recovery, and hybrid (online and offline) options for greater flexibility and widening of access and choice. Participants also expressed a need for appropriate digital literacy training.

**Discussion:**

Future recommendations emphasise user-centred holistic and hybrid approaches to ED remote support, with training to address digital literacy barriers and facilitate user control of platform functionalities (e.g., self-view). This study underscores the need for continued remote care with a focus on inclusivity and user empowerment.

## 1 Introduction

The COVID-19 pandemic severely impacted people with lived experience (PWLE) of eating disorders (EDs) (1–4). While all members of society were susceptible to the negative mental health impacts of the pandemic, PWLE of EDs were particularly vulnerable to the societal upheaval caused by COVID-19; this included mandated lifestyle changes, increased psychological stress, and enforced constraints on social support networks (5–7). A growing body of research demonstrates increased symptom severity and/or likelihood of ED diagnosis because of the pandemic (1–3). The pandemic also saw an increase in disordered eating amongst people without a formal diagnosis (8).

Amongst the challenges raised by the pandemic was the sudden transition to remote care, necessitated by lockdown (9, 10). During the initial phases of the pandemic, healthcare providers and service users were largely unprepared for delivering and receiving online treatment and support (11–13). Although remote provision was on future agendas pre-pandemic, a 2021 report by the Institute for Public Policy Research found that COVID-19 sparked the widespread adoption of remote care far sooner than the NHS had intended (14). With respect to services for EDs in the UK, neither the NHS Long Term Plan (15), NHS Long Term Workforce Plan (16), nor the NHS Mental Health Implementation Plan (17) provide specific guidance for the remote delivery of ED care. Existing evidence-based protocols and practice have largely focused on working face-to-face with patients (18). This meant that, in response to the pandemic, providers of ED services in the UK had to rapidly adapt with little formal guidance. Consequently, the pandemic has been credited for driving the transformation of healthcare delivery (19, 20). However, it is vital that we reflect on the lessons we can learn from this period, including acknowledging challenges, to further improve future provision.

Remote care can provide many benefits including improved flexibility and access. This can be particularly valuable for those who find it difficult to access face-to-face services, whether due to locality, work schedule, caring responsibilities, mobility, finances etc. (8, 21, 22). Remote care can also facilitate simultaneous access to multidisciplinary treatment teams (9). However, challenges have also been reported including inequalities in digital access and/or literacy; mistrust in technology leading to a reluctance to share information via online platforms (23); feelings of remote care becoming a burden (24); and difficulty accessing care due to personal living situation, e.g., shared households (21). There are also broader fears that the promotion of remote care by some health providers could be motivated by reduced costs associated with remote delivery, rather than by identification of the most appropriate/effective treatment method for specific conditions and/or individuals (19).

Whilst many of these concerns apply across remote healthcare more broadly, there are also more nuanced challenges around remote care for ED support, for example platforms can exacerbate self-monitoring and negative critiques of one’s perceived appearance (9, 25). This can be particularly challenging for PWLE, as dissatisfaction with one’s self-image is strongly associated with most EDs (9, 26). Remote care may also increase the burden on individuals to self-monitor their weight and ED symptoms. Open weighing can be a key component of ED treatment and safely adapting in-person protocols to remote provision is challenging (18). Remote care can also limit service providers’ ability to make best use of evidence-based treatment methods, such as exposure therapy, an important method to treat EDs (27). Clinical guidelines in the immediate response to COVID-19 highlighted that exposure therapy is much easier to deliver in-person (18).

Clinical recommendations produced during the pandemic offered a valuable resource for helping providers transition to online services, however this guidance was specific to the constraints of the pandemic with mandated lockdowns, limited options for delivery of care, and the surge in popularity of video-conferencing platforms (9). Now that online, in-person, and hybrid treatment options are available, a better understanding of the factors that influenced the effectiveness of remote care during the pandemic is needed to ensure that service users receive the most appropriate mode of treatment delivery (21). This can be achieved by investigating lived experience of receiving remote support during the pandemic and learning from these insights to improve future provision, including supporting equitable access. The current mixed methods study addresses these needs, exploring wider impact of the pandemic for PWLE of EDs, whilst capturing in-depth insights into remote support experiences (1, 2, 6). For ecological validity and to reflect the reality of care provision, it is important to consider the range of remote care interventions that PWLE of EDs accessed since the start of the pandemic (e.g., were PWLE of EDs able to access ED specific treatments remotely or did they rely on generic mental health support e.g., Talking Therapies?). This approach is important in considering what types of provision are likely to be accessible going forward, including for PWLE of EDs without a formal ED diagnosis. An assessment of the impact of the COVID-19 pandemic on ED symptomologies also allows for an assessment of the potential longer-term impact of the pandemic, and what lessons can be learnt in terms of how remote care can respond to specific needs going forward (e.g., decisions about camera self-view in video conference calls, when to consider remote care as part of a hybrid package of support etc.). A mixed methods approach was applied, combining qualitative and quantitative methods. This captures the scale of the impact of the pandemic on PWLE of EDs and the extent to which remote support was accessed (quantitative); whilst also allowing for a rich, phenomenological exploration of the factors that facilitate both challenging and effective remote care experiences (qualitative). The study objectives are therefore three-fold: i. Identify key challenges and benefits of receiving remote care for EDs during the pandemic; ii. Assess the impact of the pandemic on ED symptoms; and iii. Identify barriers to remote care and recommendations for future improvement.

## 2 Materials and methods

This study was approved by the Northumbria University Psychology Department Ethics Committee (Ref: 45202). The pre-registered study protocol, materials and anonymised data are all available on the Open Science Framework (osf.io/ucdkg/).

This sequential mixed methods study incorporates two phases: Phase 1, a series of online and in-person workshops and interviews conducted between July 2022 – January 2023. Phase 2, an online national survey conducted between January – July 2023. Phase 1 and Phase 2 were two different cohorts of participants, no participants took part in both phases. We present the methods and results from each phase sequentially, followed by a combined discussion.

## 3 Phase 1: workshops and interviews with people with lived experience of eating disorders

### 3.1 Participants and recruitment

A total of 27 participants were recruited via social media, via posters distributed across a university campus in the Northeast of England, and in close liaison with several ED charities across the UK. For safeguarding reasons, participations were screened prior to participation; individuals who were currently receiving in-patient support or who had received in-patient support within the previous six months were ineligible to participate. These safety parameters were agreed by the research team following consultation with a local ED charity. The exclusion criteria were recommended to ensure that participants were well enough to engage in the research activities. Individuals recently discharged from inpatient treatment can sometimes experience acute psychological distress and must be provided with adequate time to recover. All participants were required to complete the pre-screening survey. The survey asked if participants had a formal diagnosis and, if so, the type of ED(s) diagnosed. In the event of no formal diagnosis, the participant was asked to complete the SCOFF scale (28): a validated five-item questionnaire used to indicate the presence of a potential ED. A score of 2 or more on the SCOFF scale indicates potential likelihood of ED symptomologies. Participants who scored two or more on the SCOFF or who had a formal diagnosis were included in the study. Additional demographic information of age, gender, and ethnicity was recorded. Participants were remunerated for their time. Full sample demographics are shown in **Table 1**.

**Table 1:** Participant demographics.

	Category	Qualitative data *(n = 27)*	Quantitative data *(n = 184)*	Total study sample *(n = 211)*
**Age**	18 to 24	10 (37.04%)	21 (11.41%)	31 (14.69%)
	25 to 34	9 (33.33%)	65 (35.33%)	74 (35.07%)
	35 to 44	3 (11.11%)	47 (25.54%)	50 (23.70%)
	45 to 54	4 (14.81%)	34 (18.48%)	38 (18.01%)
	55 to 64	0	13 (7.07%)	13 (6.16%)
	65 and above	1 (3.70%)	4 (2.17%)	5 (2.37%)
**Gender**	Man	4 (14.81%)	72 (39.13%)	76 (36.02%)
	Woman	19 (70.37%)	108 (58.70%)	127 (60.19%)
	Other	3 (11.11%)	4 (2.17%)	7 (3.32%)
	Non-disclosed	1 (3.70%)	0	1 (0.47%)
**Ethnicity**	White British/Irish	18 (66.66%)	156 (84.78%)	164 (77.73%)
	White Other	3 (11.11%)	10 (5.43%)	13 (6.16%)
	South Asian	1 (3.70%)	8 (4.35%)	9 (4.27%)
	South East Asian	0	3 (1.63%)	3 (1.42%)
	Black British/Irish	0	3 (1.63%)	3 (1.42%)
	Person of Colour British/Irish	0	1 (0.54%)	1 (0.47%)
	Afro-Caribbean	0	1 (0.54%)	1 (0.47%)
	Other	5 (18.52%)	2 (1.09%)	7 (3.32%)
**ED Status**	Current eating disorder	19 (70.37%)	43 (23.37%)	62 (29.38%)
	In recovery	3 (11.11%)	34 (18.48%)	37 (17.54%)
	Struggling with disordered eating	5 (18.52%)	107 (58.15%)	112 (53.08%)
**Formal Diagnosis**	Yes	19 (70.37%)	23(29.87%)	42(19.91%)
	No	8 (19.63%)	54(70.13%)	62(29.38%)
			
Reported ED^*^	Anorexia	15 (55.56%)	12 (6.52%)	27 (12.80%)
	Bulimia Nervosa	5 (18.52%)	10 (5.43%)	15 (7.11%)
	Binge Eating Disorder	5 (18.52%)	45 (24.46%)	50 (23.70%)
	EDNOS (Eating Disorder Not Otherwise Specified)	8 (29.63%)	17 (9.24%)	25 (11.85%)
	Other	5 (18.52%)	3 (1.63%)	8 (3.79%)

*Diagnosis is not mutually exclusive, some participants reported more than one diagnosis.

### 3.2 Procedure

Using a structured interview process, PWLE of EDs were invited to take part in either a group workshop or a one-to-one interview, both of which could take place in-person or online (via Microsoft Teams). These options were offered to increase inclusivity, e.g., some individuals are more/less comfortable discussing their ED symptoms and recovery with others, likewise online and offline options cater for different individual preferences and circumstances. Workshops and interviews were conducted by a member of the research team (CMM) with additional workshops support provided by the principal investigator (DBB). Sessions were interactive with participants shown visual digital whiteboards for collaborating and sharing ideas (using Padlet, www.padlet.com). Participants were asked 3 main questions (1): *What were the key influences on your ED symptoms during the pandemic?* (2) *What types of platforms did you use during the pandemic to access support?* (3) *What types of future remote care options would you like to be offered?*

### 3.3 Analysis

A phenomenological approach was adopted to facilitate exploration of the challenges, benefits, and nuances of remote care, directly from the perspectives of recipients of remote care themselves. An inductive approach ensured that meaning, and the resulting themes, were driven by the data and not by preconceived theories. Reflexive thematic analysis (27) allowed for flexibility in the interpretation of patterns of meaning across the data set and allowed for researcher subjectivity. All workshops and interviews were recorded for transcription purposes only. One of the researchers (CMM) was responsible for data collection and coding (27). Data was manually coded, with similar codes grouped together and used as building blocks to generate initial sub-themes. Provisional final themes were generated and were shared with a second researcher (DBB) for quality assurance and to check for consistency. The approach was iterative and reflective, with themes revisited and reviewed between phases by both researchers, until a consensus was reached.

### 3.4 Results

In terms of digital platforms used to access ED advice and support, the Zoom video calling platform was the most widely used (*n*=14), followed by Microsoft Teams (*n*=10) and Text SMS (*n*=6). The most widely reported types of online or remote care accessed were Cognitive Behavioural Therapy (CBT); Nutritional Therapy; Body Image Therapy; Talking Therapy; Peer Support and local not-for-profit ED services. 2 participants reported receiving no support.

In terms of the benefits and challenges of remote care during the pandemic, the following themes were generated from the data set: (1) *Accessibility and flexibility;* (2) *Social Connection;* (3) *Challenges to creating a therapeutic environment remotely;* (4) *Masking;* (5) *Platform literacy;* and (6) *Seeing yourself on-screen.* Each theme is examined in turn.

#### 3.4.1 Accessibility and flexibility

Participants described remote support as a crucial lifeline during the pandemic, allowing access to support without having to travel (permitting individuals to isolate and/or avoiding concerns around restrictions during lockdown). Participants also reflected on remote care reducing the time needed to access support sessions, increasing convenience, and allowing flexibility:

*“It’s a lot more accessible. It [appointment] takes a lot less out of the day and you’re not travelling and hanging about.”*

Remote care also provided opportunities to synchronise and streamline existing ED services, providing, in some cases, quicker and more efficient communication between patients and healthcare providers:

*“I was able to contact my support workers and the dietitian, I could see whether they’d received the text messages sent, and they’d get back with quick responses.”*

Remote provision also resulted in ED organisations being able to increase the services they offered, such as providing support sessions which would previously have been harder to organise due to capacity constraints. The online format also increased accessibility as some sessions did not need to be delivered asynchronously, and ‘live’ sessions could be recorded and replayed for service users who were not able to attend:

*“My local ED service had ‘live’ [online support sessions] most days where they would offer general support, meal ideas, chats about ED symptoms, psychoeducation, and stuff. These sessions can then be watched back any time, so they were there when I felt I needed motivation and support.”*

Flexibility afforded by remote care could be particularly beneficial for individuals who find travel to and from face-to-face appointments challenging (e.g., due to childcare and/or dependent care responsibilities, or due to location, health and mobility, time, or financial constraints).

#### 3.4.2 Social connection

In addition to providing access to healthcare services, remote care also promoted a sense of connectedness through connection to others, including peers. This was particularly important during lockdown when feelings of social isolation were elevated:

*“It was great to meet and connect with new people.”*

For some participants, video calls provided an interaction akin to in-person interaction:

*“It [Microsoft Teams] is like face-to-face interaction. Like a phone call but easier to talk.”*

*“Through video you can see other people’s reactions and faces.”*

However, other participants had mixed experiences of social connection through remote platforms. Whilst many acknowledged that they provided a form of connection with others, for some they also keenly felt the physical distance between themselves and the other person(s) on the call:

*“When there’s a rift or an upset, it’s very hard online, especially afterwards, and no way of preparing for that in some way.”*

*“You can’t hug online!”*

Others felt that distractions can be a problem when communicating remotely:

*“People actually listen when you meet them in-person - there isn’t all the distractions [of being remote].”*

And that loss of some non-verbal communication cues could result in communication challenges:

*“I just hate video chats! It just feels awkward. You can end up talking over each other.”*

Social connection has implications post-pandemic as EDs are often comorbid with feelings of social isolation. These responses indicate that remote care can help promote feelings of social connection in some contexts. However, care must be taken to recognise that some individuals felt that remote platforms made the physical distance between themselves and others more salient, therefore potentially perpetuating feelings of isolation. This indicates the importance of customising delivery approach to the individual, with remote care considered as a complement to face-to-face support; perhaps as options within a hybrid approach.

#### 3.4.3 Challenges to creating a therapeutic environment remotely

Accessing remote care appointments from home was a double-edged sword for our participants. As aforementioned, it could be a huge benefit – affording flexibility, convenience, and improved accessibility. However, some participants experienced challenges around accessing remote care in their own home. For instance, participants talked about the importance of confidentiality and the physical space required to ensure privacy, which was not always possible due to individuals’ living arrangements. This prevented some participants from feeling comfortable enough to talk candidly with their therapist or support group:

*“In a word: confidentiality. We only have 2 rooms!”*

*“I was worried about who could hear. It was really difficult when everyone was at home.”*

There were other barriers to remote access, including relying upon home internet. Participants reported difficulties due to poor internet connection, which could have an adverse effect on their ability to communicate thoughts and feelings during remote sessions. This had implications for the whole therapeutic process, impacting on the most basic ability for effective interaction between therapist and client:

*“It’s awful if you are disclosing something and then you have to repeat it all over again!”*

*“It becomes a jolted conversation as you have the same cues, but then the internet can fade, and then it [therapy session] doesn’t follow a natural flow because of things like time delays.”*

Participants’ living circumstances were not always conducive to ensuring confidentiality. This, combined with unreliable internet connection, can result in an inferior therapeutic or supportive experience in comparison to face-to-face services. It is also important to flag that these issues can increase inequalities in access to support as the challenges may impact individuals on lower incomes and/or individuals who are not safe in their own home (e.g., coercive control). These findings suggest that face-to-face support should still be available as an option when possible.

#### 3.4.4 Masking

Participants reported that remote care could also provide unhealthy opportunities for the patient or service user to ‘mask’ how unwell they really were, encouraging secrecy and the hiding of adverse ED symptoms. This could have serious implications for the healthcare provider being able to accurately monitor an individual’s physical and mental health:

*“When you did get an appointment online, they [the therapist] couldn’t see you in person, so you could pretend that you hadn’t lost any weight and that you were sticking to the meal plan. So, it could allow the illness to lie and deceive online.”*

Service users may also mask key emotions which are not necessarily related to their ED’s but are an important part of recovery. The healthcare provider may miss key changes in body language in the service user, such as change in facial expressions, voice tone, and eye contact. Body language is often a potential indicator that an individual is struggling, not being honest, or finding it hard to engage in the session:

*“It’s so easy to lie online. To say, ‘I’m fine.’ I am not a liar, but the ED lied to me.”*

This serves as a reminder that secretive behaviours can be a strong component of EDs. The affordances of remote care platforms can exacerbate this by allowing service users or patients to ‘mask’ the true extent of their symptoms (e.g., altering camera image, switching off camera, concealing body shape). This has considerable implications for physical monitoring, compounded further by the aforementioned ethical and safeguarding challenges around remotely monitoring weight (18).

#### 3.4.5 Platform literacy

In addition to challenges with internet connection, participants talked about familiarity and literacy with online platforms. This included issues around initial setup and gaining access (e.g., how to register for accounts). Downloading apps could often be difficult to do, with little advice and guidance on how to install or use them, including how installation and screen views may differ across devices (e.g., laptop vs mobile phone). Participants talked about hurriedly attempting to familiarise themselves with platform functions so they could receive support during the pandemic. Often this involved trying to work out functions whilst already on a video call. Participants talked about their lack of knowledge about how to use, or maximise, the benefits of platform functions (e.g., chat functions, reaction options, mute-unmute, etc.):

*“I could never work out how to use the chat [function], and I could only see the person who was speaking on my tablet.”*

*“It took me a while to get used to mute and unmute – I got there in the end!”*

Online sessions could be further complicated by additional platform functions (e.g., breakout rooms) which could suddenly change the format of the platform, which some participants found disorienting. Whilst these functions can be beneficial for small group work (e.g., peer support, group CBT), they can also be daunting and confusing if individuals are left feeling unsupported. Furthermore, healthcare services do not always use the same platforms, adding another layer of anxiety when participants needed to familiarise themselves with numerous platforms. It is important that service providers also provide guidance and/or training relevant to the platforms they use, prior to remote care delivery.

#### 3.4.6 Seeing yourself on-screen

Video call platforms can seem unnatural for many users, one of the main factors in this is the self-view camera that is presented by default during calls. Many participants found this self-view distressing and worried that it could exacerbate their ED symptoms, particularly when they felt at their most vulnerable:

*“I found it difficult to see myself on video calls, particularly on bad body image days.”*

*“There’s the self-awareness thing. You can see yourself. You’re reminded of your body image.”*

For many participants this had an adverse effect as they felt unable to fully engage in the remote care process because of feeling anxiety and feeling distracted by their self-view. Additionally, even when participants were aware that they could turn their camera off (which relates back to the previous theme: *platform literacy)*, some reported still worrying about what others could or could not see:

*“I have this anxiety of knowing – is my camera on or off?!”*

Interestingly, most participants acknowledged that anxiety around viewing oneself on-screen is common. For instance, they were aware that in peer support sessions, they were not alone in experiencing this challenge. It is possible that this may offer some comfort for participants, and/or in some instances may even form part of the therapeutic process – however, this requires appropriate support and safeguarding mechanisms to be in place.

Despite self-view being a challenge shared by almost all participants, it is important to emphasise that, despite now having experience of remote platforms due to the pandemic, *none* of our participants were aware that common video call platforms (e.g., Zoom) provide options to hide self-view. Disabling self-view differs from turning off the camera (the latter could negatively impact on the effectiveness of the remote care session). When self-view is hidden, others in the meeting can still see the individual, but the individual does not have to be presented with their own self-view video. This provides a more natural environment, akin to offline interaction (where you would see others but not yourself) and could greatly improve users’ experiences of remote care by allowing them to participate with reduced stress and/or distraction.

These findings emphasise the importance of ensuring that service users are provided with clear instructions of all relevant functionalities prior to the use of remote platforms. Service providers must be aware of the potential implications for support effectiveness if the service user finds themselves distracted by self-view, and potential for distress and/or triggering of ED behaviours. This also has implications for service providers in terms of thinking about when and how they may wish to communicate the benefits of having the camera switched on, and how this can be negotiated and managed in a safe manner.

### 3.5 Future remote support recommendations

We asked participants what they would like to see regarding future remote support provision. Three themes were extracted from the data set: (1) *Functionality;* (2) *Content;* and (3) *Hybrid Options*. We look at each one of the themes in turn.

#### 3.5.1 Functionality

All participants emphasised the importance of ease of use for remote platforms and the importance of training prior to online sessions; including ensuring that assumptions are not made by service providers around users’ platform literacy:

*“I would like it laid out properly and easy to use.”*

*“More training on how to use functions would be better, both for Zoom and Teams, so people are familiar with it. So, more training and support.”*

Many participants also emphasised the importance of making it easier to make choices about camera settings from the outset:

*“Not making your own camera the default at the start of the meeting. It should be a choice, like you could go and switch it off in the settings.”*

Participants also suggested that affordances of digital platforms could be utilised further, for example by adding complementary tools or resources that could be accessed prior to, or following, therapeutic or group work sessions:

*“Can worksheets for EDs be included on a digital platform, so that anyone can access?”*

All participants reported that they would like to have easy to navigate platforms and were keen to be able to navigate *existing* platforms better. Platform training before remote sessions was widely suggested, so that they could feel better prepared and less anxious about accessing remote care. Worksheets and complementary resources for before/between sessions also point to the potential for provision of additional support using remote platforms.

#### 3.5.2 Content

Participants expressed a desire to see testimonies and stories from PWLE of EDs, including stories of recovery. They expressed the importance of these stories being relatable, non-clinical and honest; with participants feeling frustration around current online information and support lacking these real experiences. Their suggestions for future remote care included:

*“Like experience content, people can relate to real people.”*

*“People sharing their recovery.”*

*“This includes people without a diagnosis talking.”*

*“Can we not talk about stuff so clinically?!”*

There was also the perception that remote care content (e.g., websites and other online resources) could reduce patients or service users to ‘the ED condition’ as opposed to being treated as a person with varying support needs:

*“I think it should be case-by-case and not just general info. Like a holistic perspective, so like nutrition as well as psychiatric stuff.”*

Additionally, there was widely reported frustration about services not being clear enough about who the support was for (e.g., people with ED diagnoses, people without diagnosis, families, carers) and participants felt this could lead to individuals perceiving support as ‘not for them’ – providing a barrier to access:

*“To make it clear that it [online support] is for everyone who feels they might struggle.”*

Participants’ feedback is a reminder of the importance of online content. How information is presented, and who it appears to be for, can facilitate or block support access. This is also crucial for individuals without a diagnosis who may otherwise receive no information or support at all. Additionally, participants reported a lack of content that focused on the voices of PWLE of EDs in their own words, especially in the context of recovery, which is often missing from discourse about EDs. Some participants reported utilising social media, despite identifying that this meant navigating potentially triggering or inaccurate content, to find ‘real stories’ from ‘real people’ about ED recovery. These responses serve as a reminder of the lack of opportunities to access relatable content that is realistic, safe and where individuals do not need to navigate potentially triggering and/or detrimental content.

#### 3.5.3 Hybrid options

All participants were keen for remote and online care to act as a complement to in-person care and not a replacement for face-to-face services. All participants felt both in-person and remote care options were mutually beneficial:

*“In-person appointments should work in tandem with online appointments and one shouldn’t replace the other. Get the best of both worlds.”*

*“It’s important to still have the social aspect online – what if there was an event on or an open day where people could come and meet in-person and then you could catch-up virtually?”*

Some participants talked about the advantages of being able to meet in-person to build rapport with the support provider; suggesting this would be a benefit prior to online care:

*“Face-to-face interaction makes it more beneficial and it’s less difficult to open up – it makes it more personal”*

Participants’ feedback is a reminder that, whilst many of the advantages of remote care (flexibility, convenience, increase service capacity etc.) continue to make online services desirable post-COVID-19, they should not be at the expense of face-to-face services. This feedback provokes questions about the role of remote care as a complement, and not replacement, for existing face-to-face access. This is particularly crucial in the context of digital inclusion, and in continuing to provide services for whom access to digital devices is a challenge, including individuals from marginalised communities. It is also important to consider when to use remote care at different time points during an individual’s recovery journey, and when essential face-to-face monitoring is required.

## 4 Phase 2: online survey

### 4.1 Participants and recruitment

PWLE of EDs were recruited via Prolific.com and remunerated for their time. An initial screening survey recruited 310 UK adult participants. Similarly to the inclusion criteria for Phase 1, participants were screened for inclusion using either a record of a formal ED diagnosis, or for those without diagnosis, using the SCOFF scale (29). Participants with formal diagnosis or who scored two or more on the SCOFF were invited to take part in the full online survey. Our final sample consisted of 184 PWLE of EDs: 43 who self-reported as currently living with an ED, 34 in recovery, and 107 who did not report having an ED but who did report currently struggling with disordered eating. The latter was reported using the item ‘*I do not identify as having an eating disorder, but I do struggle with issues around eating, food, weight and/or exercise*’. The 107 participants who fitted into this category were included due to not wanting to limit the sample to those with a formal diagnosis only. This is important given the exponential rise in the number of sub-clinical individuals whose EDs go undetected (28, 30). Participant age, gender and ethnicity were also recorded. Full sample demographics are shown in **Table 1**.

### 4.2 Procedure

The online survey asked participants for their age, gender, ethnicity, and whether they had received a formal diagnosis. Participants were also asked which ED they reported having (‘*Anorexia Nervosa*’, ‘*Bulimia Nervosa*’, ‘*Binge Eating Disorder*’, ‘*EDNOS Eating Disorder Not Otherwise Specified*’, or ‘*Other*’). During the survey participants were also asked about the overall impact of the pandemic on their ED symptoms and/or recovery, factors positively or negatively impacting their symptoms and/or recovery, and to share details of the remote care they did, or did not, access during the pandemic (including benefits and challenges).

### 4.3 Analysis

All statistical analyses were conducted using R (31). The following packages were used for data processing, analysis, and visualisation: ggplot2 (32), psych (33), Rmisc (34), and tidyverse (35).

### 4.4 Results

As expected, given the existing literature, the pandemic had a profound effect on individuals’ ED symptoms, with the majority (71.20%, *n*=131) of participants indicating that their symptoms had worsened because of the pandemic. Of the remaining participants, 12.50% (*n*=23) reported no change in their symptoms, and 16.30% (*n*=30) reported that their symptoms had improved.

Participants reported the two factors which had the largest negative impact on their symptoms as ‘*experiencing negative emotions’* and ‘*feeling like external situations were out of your control’*. It is worth noting that this observation applied when discussing influencing factors pre- and during the pandemic. It suggests that, generally speaking (i.e., outside of a pandemic), these factors likely contribute to worsening symptoms. However, the pandemic may have exacerbated this by impacting emotions and feelings of external control. Positive impacts on ED symptoms/recovery (again pre- and during the pandemic) were associated with unsurprisingly *positive emotion, and perhaps* more insightfully, *exercise, giving support to others* and receiving *peer support.* As indicated by our qualitative data, remote care has the potential to address the latter two factors (see Section 4.4).

Contrary to our interview study, most (79.89%, *n*=147) participants in our survey reported not having received any ED care or support at all (remote or in-person) since the start of the pandemic. We explored why most participants had not accessed any support. The most common reason was that participants were not aware what support was available (32.61%, *n=*60), simply choosing not to (29.35%, *n=*54), and having too many other commitments (e.g., work, childcare; 21.20%, *n=*39).

For those who did access remote care (20.11%, *n*=37), the most reported was peer support (8.70%, *n*=16), Talking Therapy (7.61%, *n*=14), Cognitive Behavioural Therapy (CBT) (6.52%, *n*=12), and Nutritional Therapy (5.43%, *n*=10). With the exclusion of Body Image Therapy, these findings corroborate with our Phase 1 qualitative sample regarding methods of remote care accessed.

A frequency analysis of text-based responses in the optional qualitative section of the survey revealed mixed experiences of remote care, with the following 3 most frequently reported benefits and 3 challenges:

In terms of benefits, the most frequently observed comments related to: i. Connecting with others (*e.g., ‘you can talk to people while seeing them’; ‘interactions with others’* and *‘inspiration from fellow sufferers’);* ii. Access to reliable and/or wider support (*e.g., ‘being able to access a therapist when the world was shut down’* and *‘I can have access to a wider range of healthcare practitioners’);* iii. Flexibility (*e.g., ‘easy to use’* and *‘you can fit it* [remote care] *into your schedule’ and ‘The distance* [helped]. *I didn’t have to be in a room with a nutritionist but could benefit from the interaction’*).

Reported challenges included: i. Feeling more disconnected (e.g., *‘sometimes online support makes me feel disconnected and feels like there’s a barrier there that wouldn’t be there in real life meetings’*); ii. Technical difficulties (*e.g., ‘internet connection can be a factor’* and *‘not everyone has access*’) and iii. Limitations of online communication, with problems encountered because of the affordances of remote platforms themselves, and their implications for a satisfactory therapeutic experience (e.g., *‘*[You can] *hide behind the camera’* and *‘not able to pick up social cues/body language’*).

The identified benefits and challenges echo much of those described by our interview/workshop participants, with additional comments made from survey responses about accessing support from reliable and trustworthy sources.

**Figure 1:**
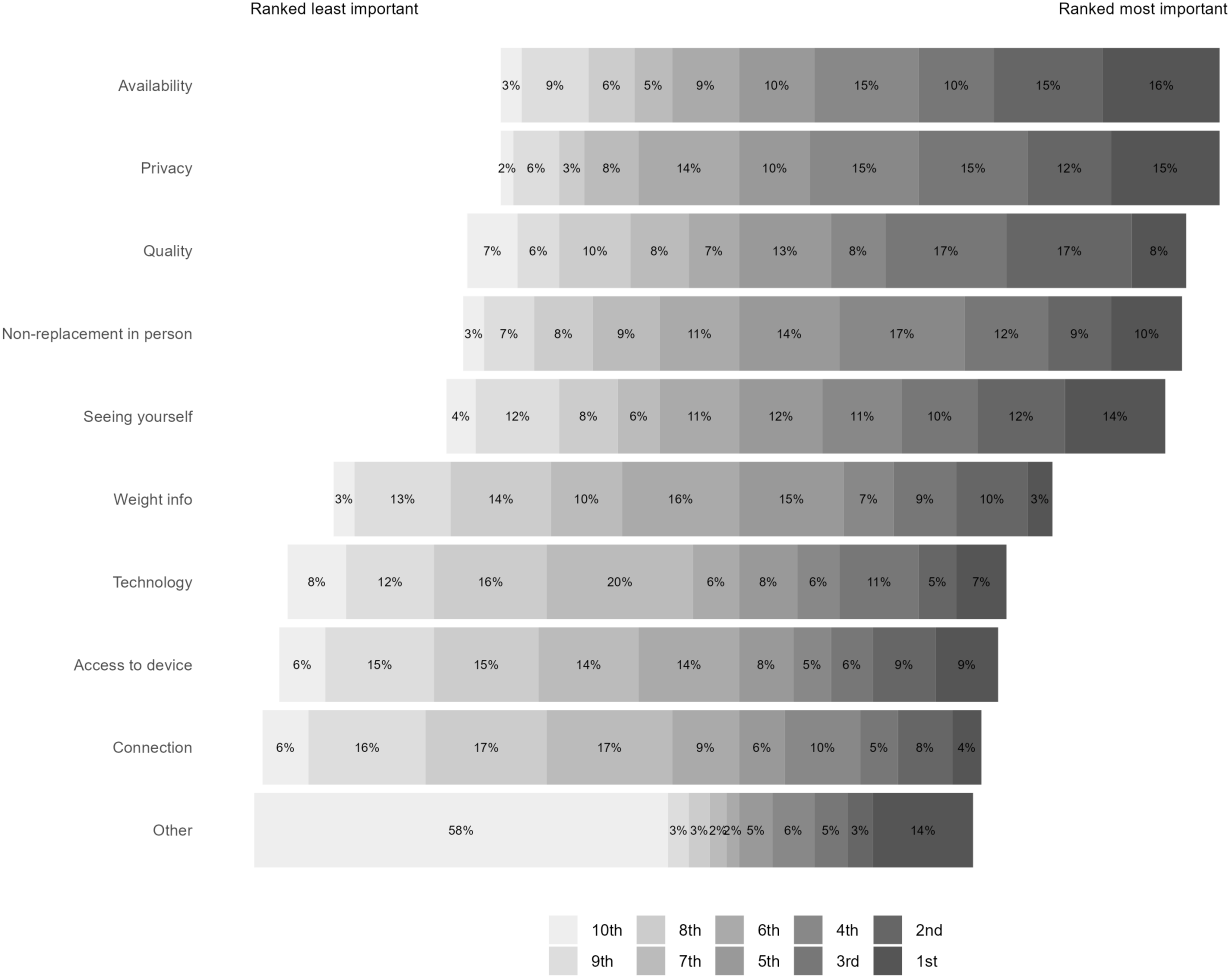
Participant rankings of the importance of different factors in preventing future access to remote care.

We asked all our participants what factors were most important for future remote support, 42% of our sample ranked privacy, availability, and quality as the three most important factors (see **Figure 1**).

## 5 Discussion

Participants across both our qualitative interviews/workshops and our quantitative survey reported a significant negative impact of the pandemic on their ED symptoms and recovery, supporting existing literature (1–3). They reported the following factors as contributing to worsening symptoms: isolation, lack of routine, negative emotions and feeling like the external situation was outside of their control. Participants reflected on access to healthcare being a significantly more negative influence on their ED symptoms during the pandemic. Whilst positive impacts were experienced because of positive emotions and giving and receiving peer support. Exercise was also identified as a coping mechanism and was recorded by survey participants as positive. However, this finding should be approached cautiously in that excessive physical activity can be a common ED symptom. The positive perception of being able to exercise can be consistent with ED symptomologies, often as a means of managing negative emotions (36). In the optional text section where participants could expand on their positive responses, they referred to permitted daily exercise during lockdown as examples of why they found exercise a positive experience during the pandemic. Whilst it is possible that individuals were exercising in a healthy manner, it is outside the scope of these findings to identify to what extent participants understood healthy or unhealthy levels of exercise. It is also important that exercise during this period is interpreted in the context of other aspects of life during the pandemic – many of which may impact upon the decision to exercise and/or its perception as a positive or negative factor. For example, there was increased incarceration at home (including potential for heightened family conflict), and greater time spent on social media (potentially resulting in intensified focus on health and fitness (22). Existing research reported PWLE of EDs engaging in increased physical activity during lockdown to compensate for calories consumed; even though they were receiving ongoing remote support for their ED(s) (37, 38). Greater trait intolerance of uncertainty has also been associated with compulsive exercise during the pandemic (39). There is a wealth of research demonstrating the negative impact of unhealthy exercise for PWLE of EDs, however conversely, exercise has also been suggested to play a restorative role in ED recovery (37, 40). This is not an easy issue to navigate as it can be difficult for PWLE of EDs, and clinicians, to determine where the blurred line between healthy and unhealthy exercise lies (41) and careful monitoring is important (42). Where exercise is considered beneficial, research tends to advise that these should be mindful, non-competitive, social forms of exercise, rather than those that require commitment, are particularly physically demanding, and/or promote competition (37).

In contrast to our workshops and interviews data, the majority (80%) of our survey participants had *not* accessed remote support during the pandemic (in contrast with only 30% of our interview and workshop participants). It is noted that this difference between the phases may be partially due to most of our interview/workshop participants having a formal diagnosis and/or being recruited via ED charities and services directly and therefore more likely to be accessing services. We also opened our survey to individuals without formal diagnosis (using the SCOFF scale as an indicator of ED symptoms). This decision was made to aid inclusivity as research shows an exponential rise in the number of sub-clinical individuals whose EDs go undetected (28, 30). The survey data suggests that, although ED services underwent a rapid transition to online delivery in response to COVID-19, many people did *not* access remote care during the pandemic. From our sample, the most common reasons were not knowing online support was available and choosing not to engage. This suggests that greater efforts are needed to publicise the availability of remote care services for EDs in the UK; and highlights the need to identify perceived benefits and limitations of remote care to identify how to encourage support engagement.

For those who accessed support, the most frequently reported types across Phase 1 and Phase 2 samples included CBT, Nutritional Therapy, Talking Therapy and Peer Support. The reported quality of support was varied and highly dependent on the digital resources that the individual participant had at their disposal (e.g., devices, internet connection). Effective engagement was dependent on knowledge around how to navigate and maximise use of online platforms. Service users (across both our interview/workshop and survey participants) reflected on a range of benefits and challenges. Increased service access and flexibility was a standout benefit of remote support, including the ability to access appointments from a place and at a time that was most convenient for the patient or service user; and the opportunity for service providers to offer broader services due to increased capacity afforded by digital delivery. The findings point to the potential remote care has when it comes to widening support access, and how it has the potential to be tailored to meet a range of needs, including for participants where travel to and from face-to-face appointments is a barrier, and/or for individuals with limited time (e.g., due to work/childcare responsibilities). It also has the potential to reach individuals with mental and/or physical health comorbidities for whom leaving home is a challenge.

Another key benefit identified across both data sets was the application of remote platforms as a means of social connection. This is consistent with literature exploring peer support online (43, 44). It is worth noting that some participants reported mixed experiences around social connection and remote platforms, with some reflecting that they became more aware of the physical distance between themselves and others – this seemed particularly salient when individuals expressed a desire for physical connection (e.g., wanting to hug) or when ending the call and filling the void of suddenly being alone. This suggests that there may be avenues for future research which could look at remote/hybrid interventions to expand feelings of closeness and/or to reduce or manage the potential for remote care recipients being left alone at home with difficult emotions immediately after a remote appointment or support session has ended. Travel to and from a face-to-face appointment can allow for invaluable emotional, psychological, and physical transition time between a therapy or group support session and the return to daily pressures (e.g., work, childcare etc.). Remote care, and the sudden transition from therapeutic environment to being alone at the click of a button, represents a unique challenge when it comes to considering after-support.

Other challenges included concerns around confidentiality and access (including access to devices, digital literacy and/or poor internet connection) and concerns around remote care providing increased ability to mask some ED symptoms (e.g., weight loss). This demonstrates the complexities of being able to safeguard the benefits of therapeutic interventions remotely, corroborating with previous literature (8, 18). Service providers must consider how migration to remote support may impact on individuals for whom safety and confidentiality at home cannot be guaranteed. This is similarly the case for digital access and internet connectivity. The ability to ‘mask’ severe symptoms on remote platforms also highlights the need to effectively and safely consider when it may or may not be appropriate to offer remote support to a patient or service user, and to offer remote support only after considering when face-to-face services are essential for physical monitoring. This is particularly relevant given the often-secretive nature of EDs. This suggests that further research is needed to explore how remote and in-person monitoring can be effectively combined for ED support/recovery beyond the COVID-19 pandemic.

There were also unanimous concerns around distraction, distress and/or triggering of ED symptoms caused by being presented with self-view on video calls. Participants reported that self-view allowed for continuous self-monitoring (which is in keeping with findings from the literature (22) and that disabling this feature could be beneficial to the therapeutic process. This raises questions for service providers around how to mitigate potential risk (e.g., this could include providing information around how to disable self-view) and considerations for platform developers around what options they select as the default (as self-view is the current default on all mainstream video calling platforms).

We found that participants were keen to improve their platform literacy when accessing remote support. Participants reported a lack of training opportunities in familiarising themselves with remote platforms *before* remote appointments, which presented an additional level of anxiety for in the lead-up to and during online sessions. These key points raise concerns about service providers making general assumptions about digital literacy. Participants talked about how remote platforms could help to generate complementary resources for remote care, e.g., worksheets, instructions, and guidance. Remote and digital services also potentially allow ED service providers to enhance their existing resources for advice and support, including recordings of online therapy sessions, and downloadable templates for pre- and post- session reflection. Further research using co-design approaches with PWLE of EDs could explore how these benefits could be maximised.

As identified in a recent review (3), most research in this space relies on self-report data. Self-reported data affords exploration of lived experience and is regarded as a benefit rather than a limitation. That said, we recognise that we relied upon retrospective self-report. In this instance, this method was necessitated due to the nature of the work (i.e., the unforeseen pandemic necessitating retrospective identification of pre-pandemic factors), however future research should aim to limit retrospective reflection to mitigate against inaccuracies in recall. Furthermore, we recognise that participants self-reported their ED status, i.e., whether they had a formal diagnosis, were in recovery or currently struggling with disordered eating. Whilst the inclusion criteria required a formal diagnosis or a score of ≥2 on the SCOFF scale, there is arguably still subjectivity in how individuals view and experience their symptoms. We appreciate that this raises challenges around replication. The existing data is also limited in relation to pre-pandemic care; whilst some information about the impact on previous care was recorded in Phase 1, this was insufficient to draw firm comparisons between care accessed pre- and during the pandemic. The authors recommend further research to investigate the impacts of changes and/or disruption to care during and following the pandemic, including investigation of any long-term impacts.

The study sample was purposefully non-clinical. Whilst this is an important and growing population experiencing ED symptomologies, it is important to stress that there are also limitations in terms of comparing subclinical participants with their diagnosed counterparts. Many participants reporting disordered eating symptoms may well be at the threshold of ED symptoms, as opposed to a fully developed illness. Further investigation is required to consider what remote care needs may be specific to subclinical individuals, and what remote early intervention care measures may be beneficial in aiding timely recovery.

There are valuable implications that can be drawn from this study’s findings in relation to both remote care for EDs specifically (and the unique challenges raised) and in relation to remote healthcare and support more widely. For video conferencing, the default camera settings are switched to ON for most platforms, and none of the participants interviewed during Phase 1 were aware that meeting options allow them to switch off the self-view setting. There are multiple factors which may make self-view challenging for many users across a wide range of contexts. However, the high incidence of self-monitoring and negative body image can make this particularly problematic in relation to ED support. Furthermore, remote platforms can increase the potential for masking symptom severity, including not only physical but also emotional and psychological symptoms – again there are implications for this both within and outside of ED support specifically. Even outside of any deliberate masking of symptoms, the lack of in-person monitoring can increase the potential for worsening symptoms to be missed (e.g., rapid weight decline). These factors have important implications for how clinicians and therapists determine when remote care can be used, and when in-person support is the only safe option. On a positive note, remote care can potentially act as a valuable addition, and not a replacement for in-person care. Benefits include extending available care and providing alternative support to individuals without diagnosis and PWLE of EDs experiencing other comorbidities or situational factors which make regular in-person treatment difficult.

The current study was designed to capture lived experiences of remote care from the perspective of the recipients and is not intended to examine the effectiveness of remote care. The authors recommend future research includes comparative analysis of in-person and remote care for EDs to assess effective post-pandemic implementation and identification of effective hybrid support solutions. Following the pandemic, we expect remote care to persistently maintain higher delivery rates compared to before COVID-19 (11). In the post-pandemic world, remote care is not driven by lockdown measures, and it is likely that it may now be driven by other needs or preferences (e.g., flexibility, removal of need to travel to appointments). Further research, replicating the phenomenological approach used in the current study, can provide insight into this evolution of user needs, which in turn can help to design more effective remote care solutions. Recommendations for sustainability beyond the pandemic highlighted the need for discipline-specific guidelines around remote care (11). The NHS is yet to provide clear guidance on how best to deliver remote support for ED services in the UK (15–17). Clear strategies and guidance are required, which must be rooted in research and clinical expertise. This study contributes to the required knowledge by highlighting lessons learned during the pandemic and emphasising the importance of involving PWLE of EDs in these processes. The benefits, challenges and user requirements identified in this research have implications for future service provision, policy, and guidance.

## Data availability statement

The datasets presented in this study can be found at https://osf.io/ucdkg/.

## Ethics statement

The studies involving humans were approved by Northumbria University Psychology Department Ethics Committee (Ref: 45202). The studies were conducted in accordance with the local legislation and institutional requirements. The participants provided their written informed consent to participate in this study.

## Author contributions

CM-M: Conceptualization, Data curation, Formal analysis, Investigation, Methodology, Project administration, Resources, Validation, Writing – original draft, Writing – review & editing. RB: Formal analysis, Writing – original draft, Writing – review & editing. CL: Formal analysis, Writing – original draft, Writing – review & editing. DB-B: Conceptualization, Data curation, Formal analysis, Funding acquisition, Investigation, Methodology, Project administration, Resources, Supervision, Validation, Visualization, Writing – original draft, Writing – review & editing.
